# Multi-omics identification of key targets for the osteogenic differentiation of human bone marrow mesenchymal stromal cells under oxidative stress

**DOI:** 10.1038/s41598-026-39818-4

**Published:** 2026-02-10

**Authors:** Wentao Dong, Yangyang Zheng, Yongfang Zhou, Tao Wang, Jiexin Yang, Huaying Li, Fanchao Li, Chuan Wang, Liang Liang, Hao Li, Jian Zhang, Wuxun Peng

**Affiliations:** 1 Emergency Department, The Affliated Hospital of Guizhou Medical University, Guiyang, 550004 Guizhou China; 2 School of Clinical Medicine, Guizhou Medical University, Guiyang, 550004 Guizhou China; 3 Department of Orthopedics and Emergency, The Affiliated Hospital of Guizhou Medical University, Guiyang, 550004 Guizhou China; 4 Department of orthopedic, Guizhou Provincial People’s Hospital, Guiyang, 550002 Guizhou China

**Keywords:** Bone marrow mesenchymal stromal cells, Osteogenic differentiation, Transcriptomics, Proteomics, Oxidative stress, Cell biology, Computational biology and bioinformatics, Stem cells, Medical research

## Abstract

Human bone marrow mesenchymal stromal cells (hBMSCs) are multipotent stromal cells capable of osteogenic differentiation, making them a promising cell source for bone tissue engineering and regenerative medicine. Identifying key factors that regulate hBMSCs osteogenic differentiation is crucial for enhancing bone regeneration strategies. This study aims to identify target genes underlying impaired osteogenic differentiation of hBMSCs under oxidative stress (OS) through integrated transcriptomic and proteomic approaches, and delineate the role of proenkephalin (PENK) in this process. OS model and impaired osteogenic differentiation model were established in hBMSCs using hydrogen peroxide (H_2_O_2_). Cellular oxidative stress levels were assessed using Dihydroethidium (DHE) fluorescent probes and JC-1 staining. Osteogenic differentiation was evaluated by alkaline phosphatase (ALP) activity and Alizarin Red staining (ARS). Key genes and proteins were predicted via integrated transcriptomic and proteomic analyses. The role of PENK in osteogenic differentiation was validated using lentiviral transfection. This study established 400 µM H_2_O_2_ as the optimal concentration for inducing impaired osteogenic differentiation in hBMSCs. Integrated transcriptomic and proteomic analysis identified 18 pivotal regulatory genes that orchestrate impaired osteogenic differentiation of hBMSCs under OS. Among these, PENK was identified as a potential therapeutic target involved in regulating oxidative stress-impaired osteogenic differentiation of hBMSCs. Functional validation confirmed that PENK overexpression promotes osteogenic differentiation in hBMSCs. OS contributes to impaired osteogenic differentiation in hBMSCs. PENK regulates osteogenic differentiation of hBMSCs under OS and holds promise as a novel therapeutic target for bone regeneration and repair.

**Supplementary Information:**

The online version contains supplementary material available at10.1038/s41598-026-39818-4.

## Introduction

Bone marrow mesenchymal stromal cells (BMSCs) have the capacity for self-renewal and differentiation into various cell types, including osteoblasts, chondrocytes and fibroblasts^1–3^. They also secrete a diverse array of cytokines, modulate the proliferation and differentiation of vascular endothelial cells and osteoblasts, and contribute to the repair of diseased tissues^4–6^. Human BMSCs (hBMSCs) transplantation technology is gaining traction in orthopedics, where it demonstrates significant potential. However, the osteogenic differentiation of hBMSCs is functionally impaired in adverse microenvironments characterized by ischemia, hypoxia, oxidative stress (OS), and inflammation. The precise molecular mechanism by which OS impairs osteogenic differentiation in hBMSCs remains elusive. Consequently, establishing a robust model of oxidative stress-impaired osteogenic differentiation in hBMSCs and conducting in-depth analysis of the regulatory mechanisms underlying OS-induced differentiation failure are critically essential.

OS arises from an imbalance between oxidant production and antioxidant defense mechanisms^7^. It is commonly associated with pathological conditions such as inflammation, hypertension, diabetes, aging, and neurodegenerative disorders. Elevated levels of reactive oxygen species (ROS), alongside reduced antioxidant defenses, contribute to diseases affecting the cardiovascular, endocrine, and skeletal systems^8–11^. ROS are highly unstable and reactive molecules^12,13^, their excessive accumulation leads to OS, disrupting cellular function and promoting disease pathogenesis. However, the underlying causes of the impaired osteogenic differentiation of hBMSCs under OS conditions remain unclear and require further investigation.

Together, the transcriptomic and proteomic analyses in this study offer foundational evidence to support subsequent research, highlighting promising targets and providing a basis for understanding the antioxidant stress responses and osteogenic differentiation of hBMSCs. Our multi-omics landscape reveals proenkephalin (PENK) as a redox-sensitive osteogenic regulator, with functional studies nominating it as a therapeutic target for counteracting OS-induced bone formation failure.

## Results

### Establishment of an OS model in hBMSCs

**Fig. 1:**
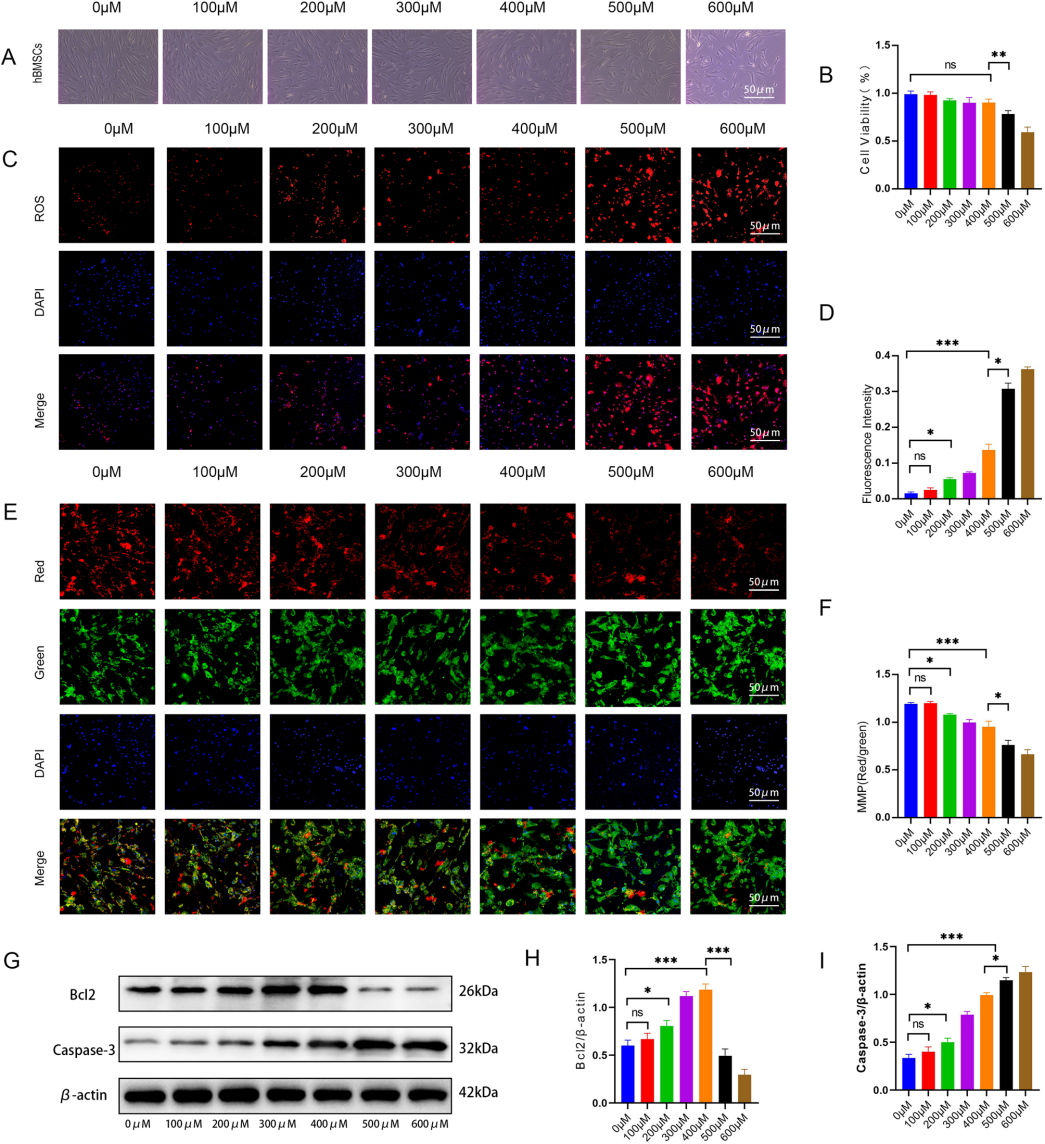
Hydrogen peroxide-mediated induction of OS in hBMSCs. **A**. Growth status of hBMSCs under varying H_2_O_2_ concentrations (*n* = 3). **B**. Cellular viability analyses performed using a CCK-8 assay (*n* = 3). **C**,** D**. Intracellular ROS detection by DHE staining (*n* = 5). **E**,** F**. MMP detection using JC-1 staining (*n* = 5). **G–I**. The expression levels of Bcl2 and Caspase-3 were detected by western blotting (*n* = 3). hBMSCs: human bone marrow mesenchymal stromal cells; OS: oxidative stress; ROS: reactive oxygen species; DHE: Dihydroethidium; MMP: mitochondrial membrane potential; BCL: 2B-cell lymphoma-2; Caspase-3: cysteine-requiring aspartate protease 3. Data are shown as the mean ± SD. *P < 0.05, **P < 0.01, ***P < 0.001, NS, not significant, by one-way ANOVA.

To evaluate the effects of OS on hBMSCs, cells were treated with a gradient of hydrogen peroxide (H_2_O_2_) concentrations (0, 100, 200, 300, 400, 500, and 600 µM). At concentrations ≤ 400 µM, hBMSCs retained their typical spindle-shaped morphology. In contrast, at ≥ 500 µM, cell density decreased and cells lost their normal morphology (Fig. 1A). Cell viability assays (CCK-8) showed no significant changes at ≤ 400 µM, but viability declined markedly at ≥ 500 µM (Fig. 1B). In addition, the isolated hBMSCs were fully characterized in vitro. They exhibited typical fibroblast-like spindle-shaped morphology and expressed high levels of widely accepted mesenchymal stromal cell markers (CD90 and CD105). Consistent with standard criteria for culture-expanded MSCs, they were negative for the pan-hematopoietic marker CD45 and, after culture, were also negative for CD34 (Supplementary Fig. 1A–E). It is noted that while CD34 is expressed by certain hematopoietic progenitors and non-hematopoietic stromal cells in vivo, its expression is typically lost upon in vitro culture of MSCs^14,15^. Furthermore, they demonstrated the capacity for tri-lineage differentiation into osteoblasts, adipocytes, and chondrocytes under appropriate induction conditions (Supplementary Fig. 1F–I).

Dihydroethidium (DHE) fluorescence staining revealed a moderate increase in intracellular ROS at H_2_O_2_ concentrations ≤ 400 µM, while significantly elevated ROS levels were observed at concentrations > 400 µM (Fig. 1C, D). JC-1 staining revealed no discernible decrease in mitochondrial membrane potential at H_2_O_2_ concentrations ≤ 400 µM, whereas a significant depolarization occurred at concentrations > 400 µM (Fig. 1E, F). Analysis of apoptotic markers revealed that at H_2_O_2_ concentrations ≤ 400 µM, both the anti-apoptotic protein BCL2 and the pro-apoptotic protein Caspase-3 exhibited responsive upregulation, with BCL2 demonstrating a more pronounced increase than Caspase-3. At H_2_O_2_ concentrations > 400 µM, expression of the anti-apoptotic protein BCL2 was significantly suppressed, while the pro-apoptotic protein Caspase-3 exhibited marked upregulation (Fig. 1G–I).

These findings demonstrate that H_2_O_2_ concentrations ≤ 400 µM maintain cell viability while inducing appropriate levels of ROS and mitochondrial membrane potential in hBMSCs. Thus, we established an impaired osteogenic differentiation model using ≤ 400 µM H_2_O_2_ for subsequent experiments.

### Establishment of an impaired osteogenic differentiation model in hBMSCs

**Fig. 2:**
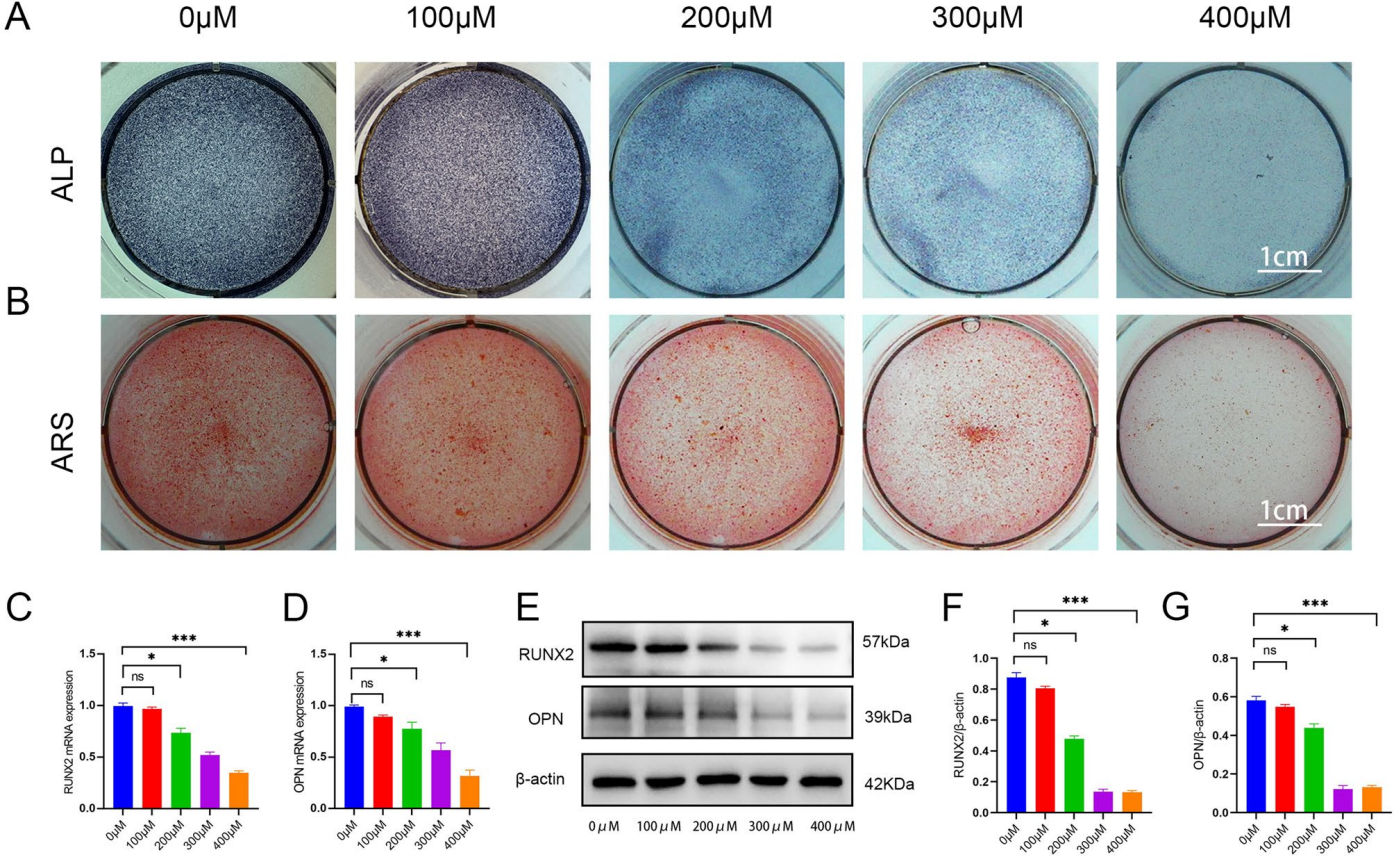
Hydrogen peroxide-induced modeling of impaired osteogenic differentiation model in hBMSCs. **A.** ALP staining reveals suppressed enzymatic activity with increasing H_2_O_2_ concentrations (*n* = 3). **B**. ARS staining reveals diminished calcified nodules with increasing H_2_O_2_ concentrations (*n* = 3). **C**,** D**. The expression levels of RUNX2 and OPN were detected by RT-qPCR (*n* = 3). **E–G**. The expression levels of OPN and RUNX2 were detected by western blotting (*n* = 3). hBMSCs: human bone marrow mesenchymal stromal cells; ALP: alkaline phosphatase; ARS: alizarin red S staining; RT-qPCR: real-time quantitative polymerase chain reaction; OPN: osteopontin; RUNX2: Runt-related transcription factor 2. Data are shown as the mean ± SD. *P < 0.05, **P < 0.01, ***P < 0.001, NS, not significant, by one-way ANOVA.

To further investigate the impact of OS on osteogenic differentiation of hBMSCs, cells were treated with 0, 100, 200, 300, or 400µM H_2_O_2_ and subsequently induced for osteogenic differentiation. Alkaline phosphatase (ALP) staining demonstrated progressively diminished intensity with increasing H_2_O_2_ concentrations, indicating reduced ALP enzymatic activity (Fig. 2A). Alizarin Red staining (ARS) staining revealed a concentration-dependent decrease in calcium salt deposition (Fig. 2B). RT-qPCR and Western blotting analyses further confirmed declining expression levels of osteogenic markers Runt-related transcription factor 2 (RUNX2) and osteopontin (OPN) in response to escalating H_2_O_2_ exposure (Fig. 2C–G). These results demonstrate that H_2_O_2_ concentrations ≤ 400 µM significantly impair osteogenic differentiation of hBMSCs, with progressively exacerbated impairment observed at higher concentrations. Therefore, we selected 400 µM H_2_O_2_ to establish a model of impaired osteogenic differentiation in hBMSCs.

### Transcriptomic profiling of OS-induced impaired osteogenesis

**Fig. 3:**
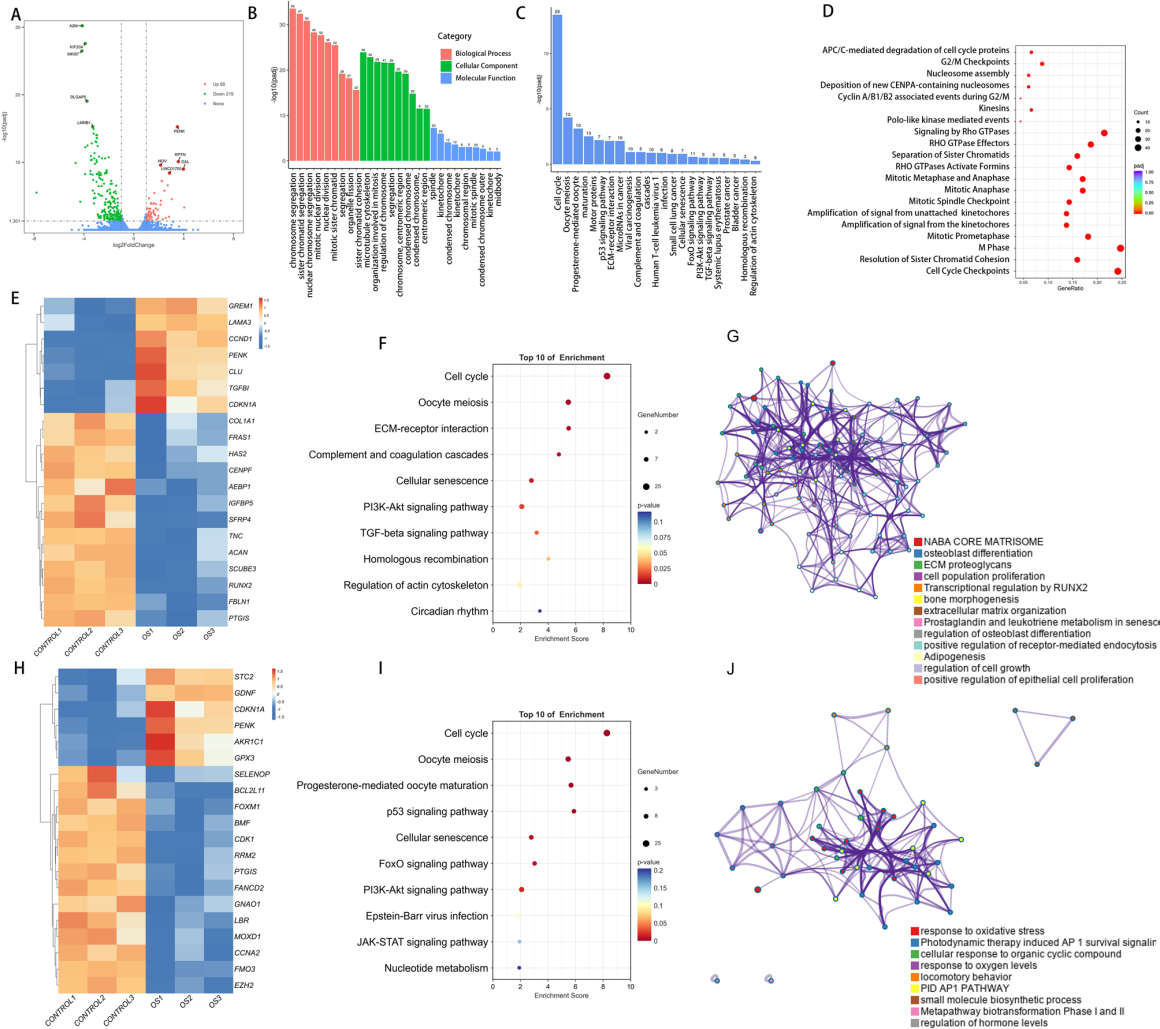
Transcriptomic analysis identifies DEGs in hBMSCs under OS **(A)** Volcano plot of RNA-seq data from hBMSCs under OS (*n* = 3). **(B)** GO analysis reveals the most significantly enriched terms (*n* = 3) **C**, F, I. KEGG enrichment analysis reveals significantly altered pathways (*n* = 3). **D**. Reactome enrichment analysis of DEGs (*n* = 3). **E**,** H**. Heatmap of differentially expressed genes associated with osteogenic differentiation and oxidative stress (*n* = 3). **G**,** J**. Topological representation graph of enriched functions (*n* = 3). DEGs: differentially expressed genes; OS: oxidative stress; hBMSCs: human bone marrow mesenchymal stromal cells; GO: gene ontology; KEGG: kyoto encyclopedia of genes and genomes.

To elucidate the potential molecular mechanisms contributing to the impairment of osteogenic differentiation in hBMSCs under OS conditions, we established a model of impaired osteogenic differentiation using these cells. In addition to in vitro experiments, hBMSCs were also isolated from clinical patients. The baseline clinical information of the enrolled patients (*n* = 6) is summarized in Supplementary Table 1. RNA sequencing (RNA-seq) analysis of hBMSCs under OS identified 284 differentially expressed genes (DEGs) (|log₂FC| ≥ 1, *P* < 0.05), including 65 upregulated and 219 downregulated genes(Fig. 3A). The quality of the RNA-seq data was evaluated comprehensively. Reproducibility across biological replicates was assessed using Pearson’s correlation coefficients (Supplementary Fig. 2 A). Principal component analysis (PCA) confirmed clear clustering of replicates (Supplementary Fig. 2B), and the distribution of gene expression levels across samples was visualized using boxplots (Supplementary Fig. 2 C). Gene Ontology (GO) analysis showed enrichment in chromosome segregation, centromeric region activity, microtubule binding, and motor activity (Fig. 3B). Kyoto Encyclopedia of Genes and Genomes (KEGG) pathway analysis further revealed significant enrichment in cell cycle, oocyte meiosis, progesterone-mediated oocyte maturation, motor proteins, p53 signaling pathway, ECM-receptor interaction (Fig. 3C). Reactome pathway analysis identified 88 significantly enriched pathways, primarily related to mitotic regulation, including cell cycle checkpoints, resolution of sister chromatid cohesion, M phase progression, mitotic prometaphase, kinetochore signal amplification, spindle checkpoint control, mitotic anaphase, metaphase–anaphase transition, and RHO GTPase-mediated formin activation (Fig. 3D). These findings indicate that the affected gene set is closely associated with mitotic progression, chromosome dynamics, and cytoskeletal reorganization.

We then focused on DEGs associated with osteogenic differentiation, including GREM1, LAMA3, CCND1, PENK, CLU, TGFBI, CDKN1A, COL1A1, FRAS1, HAS2, SCUBE3, CENPF, AEBP1, FBLN1, RUNX2, IGFBP5, SFRP4, PTGIS, TNC, and ACAN (Fig. 3E). KEGG pathway analysis showed that these genes were primarily enriched in cell cycle regulation, oocyte meiosis, ECM–receptor interaction, complement and coagulation cascades, cellular senescence, the PI3K-Akt signaling pathway, and the TGF-beta signaling pathway (Fig. 3F). GO enrichment network analysis using Metascape revealed three major functional modules: (1) core regulatory networks of osteogenic differentiation (e.g., RUNX2 regulation and bone morphogenesis), (2) ECM remodeling (matrisome components and ECM organization), and (3) regulation of cell proliferation (Fig. 3G).

In parallel, we identified OS-associated DEGs, including AKR1C1, BCL2L11, BMF, CCNA2, CDK1, CDKN1A, EZH2, FANCD2, FMO3, FOXM1, GDNF, GNAO1, GPX3, LBR, MOXD1, PENK, PTGIS, RRM2, SELENOP, and STC2 (Fig. 3H). These genes were predominantly enriched in pathways related to the cell cycle, oocyte meiosis, progesterone-mediated oocyte maturation, p53 signaling, cellular senescence, the FoxO and PI3K-Akt signaling pathways, Epstein–Barr virus infection, JAK-STAT signaling, and nucleotide metabolism (Fig. 3I). GO enrichment clustering by Metascape revealed four major modules: (1) OS sensing (response to OS), (2) AP-1 survival signaling (e.g., photodynamic therapy-induced signaling), (3) metabolic detoxification (Phase I/II biotransformation), and (4) systemic coordination (hormone level regulation and locomotory behavior) (Fig. 3J).

### Proteomic profiling of OS-impaired osteogenesis

To explore the proteomic changes associated with OS-induced impairment of osteogenic differentiation in hBMSCs, we performed 4D label-free quantitative proteomic analysis. The reproducibility of protein quantification was validated using Pearson’s correlation coefficients across biological replicates (Supplementary Fig. 2G). Data quality was assessed comprehensively through several metrics: the distribution of identified peptide ion scores, the number of identified peptides per protein, and the relative molecular weight distribution of detected proteins (Supplementary Figs. 2D–F). Additionally, PCA demonstrated clear clustering of biological replicates, and relative standard deviation boxplots confirmed the consistency of protein quantification across samples (Supplementary Fig. 2H–I).

**Fig. 4:**
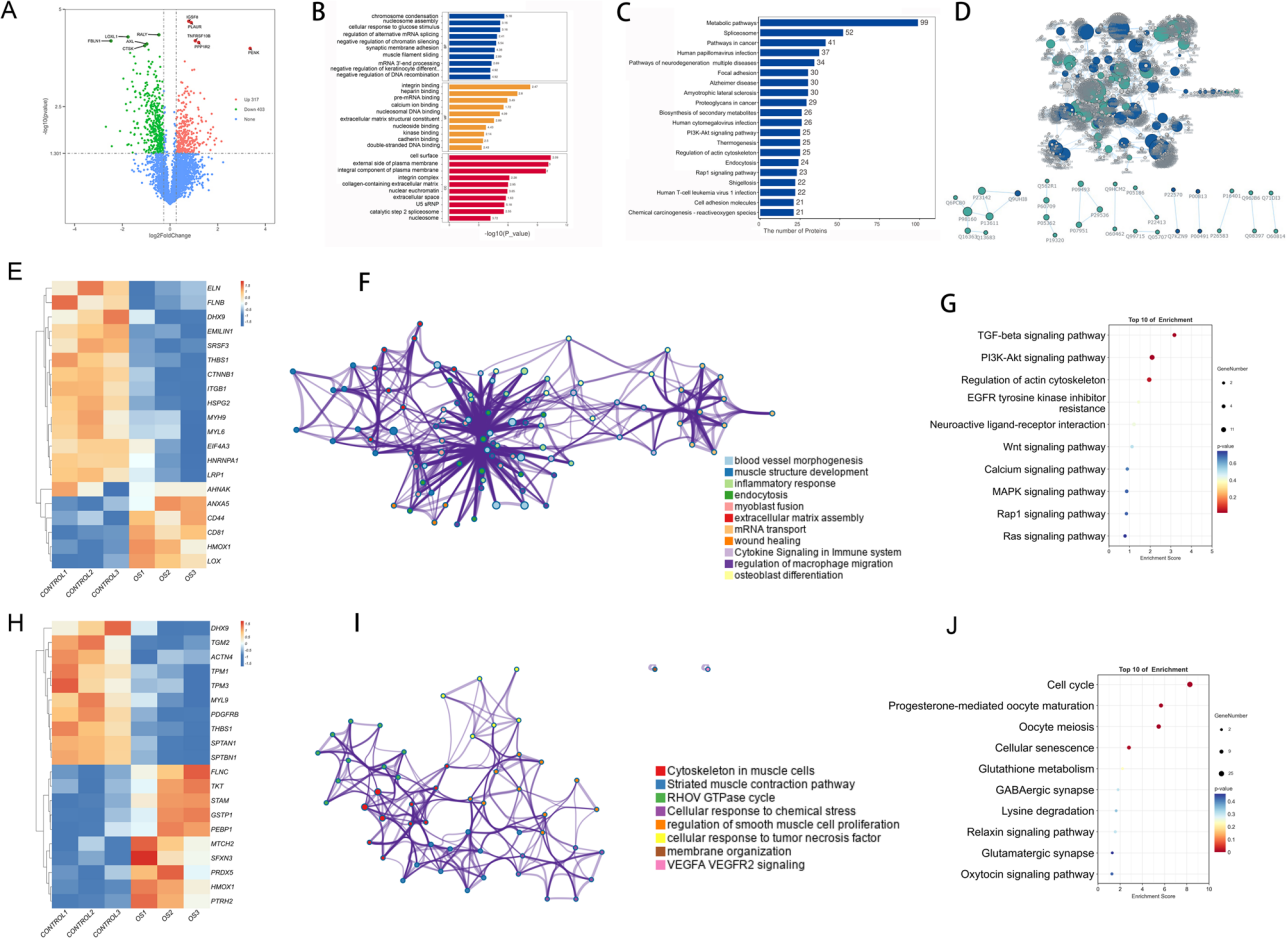
Proteomic analyses of hBMSCs subjected to OS modeling. **A**. Volcano plot displaying DEPs in hBMSCs under OS (*n* = 3). **B**. GO enrichment analysis of DEPs (*n* = 3). C. KEGG enrichment reveals DEPs association with key pathways (*n* = 3). **D**. PPI network analysis of DEPs (*n* = 3). **E**, H. Heatmap of DEPs associated with osteogenic differentiation and oxidative stress (*n* = 3). **F**, I. GO enrichment using Metascape reveals functional terms for DEPs related to osteogenic differentiation and oxidative stress (*n* = 3). **G**,** J**. KEGG enrichment reveals DEPs associated with osteogenic differentiation and oxidative stress (*n* = 3). hBMSCs: human bone marrow mesenchymal stromal cells; OS: oxidative stress; DEPs: differentially expressed proteins; GO: gene ontology; KEGG: kyoto encyclopedia of genes and genomes; PPI: protein-protein interaction.

A total of 4,293 proteins were identified, among which 720 were differentially expressed proteins (DEPs) (fold change ≥ 1.2, *P* < 0.05), including 317 upregulated and 403 downregulated in the OS group (Fig. 4A). GO analysis revealed significant enrichment in biological processes and molecular functions such as chromosome condensation, nucleosome assembly, cellular response to glucocorticoid stimulus, integrin binding, heparin binding, pre-mRNA binding, and components of the plasma membrane (Fig. 4B). KEGG pathway enrichment indicated that DEPs were primarily involved in metabolic pathways, spliceosome function, cancer-related pathways, human papillomavirus infection, and multiple neurodegeneration-associated pathways (Fig. 4C).

Protein–protein interaction (PPI) network analysis identified 30 core hub proteins, including VWA1, ITGA7, VCAM1, TPM2, PLXNA4, COL14A1, VCAN, ALPL, ACTB, FDXR, COL12A1, ENPP1, ACTBL2, COX15, LAMA4, ADA, ICAM1, ADAMTS1, HMGB2, H1-5, LOXL1, H3C15, PNP, TPM1, LMOD1, H2BC12, HSPG2, LOXL4, NRP2, and FBLN1 (Fig. 4D).

Next, we focused on DEPs associated with osteogenic differentiation, including ELN, FLNB, DHX9, EMILIN1, SRSF3, THBS1, CTNNB1, ITGB1, HSPG2, MYH9, MYL6, EIF4A3, HNRNPA1, LRP1, AHNAK, ANXA5, CD44, CD81, HMOX1, and LOX (Fig. 4E). GO enrichment network analysis using Metascape revealed three major functional modules: (1) tissue morphogenesis, including blood vessel morphogenesis, muscle structure development, myoblast fusion, and extracellular matrix (ECM) assembly; (2) immune coordination, encompassing inflammatory response, cytokine signaling in the immune system, and regulation of macrophage migration; and (3) bone differentiation, including osteoblast differentiation, wound healing, and ECM assembly (Fig. 4F). KEGG pathway analysis showed that these osteogenesis-related DEPs were significantly enriched in the TGF-beta signaling pathway, PI3K-Akt signaling pathway, and regulation of the actin cytoskeleton (Fig. 4G).

We also identified DEPs associated with OS regulation, including DHX9, TGM2, ACTN4, TPM1, TPM3, MYL9, PDGFRB, THBS1, SPTAN1, SPTBN1, FLNC, TKT, STAM, GSTP1, PEBP1, MTCH2, SFXN3, PRDX5, HMOX1, and PTRH2 (Fig. 4H). GO enrichment network analysis categorized these OS-related proteins into four main functional modules: (1) muscle structure and contraction, involving cytoskeletal organization in muscle cells and striated muscle contraction pathways; (2) cellular stress response, including responses to chemical stress and tumor necrosis factor; (3) signal regulation, featuring pathways such as the RHOV GTPase cycle, VEGFA–VEGFR2 signaling, and regulation of smooth muscle cell proliferation; and (4) cellular homeostasis, focusing on membrane organization and maintenance (Fig. 4I). KEGG pathway analysis showed that these OS-related DEPs were significantly enriched in the cell cyle, progesterone-oocyte maturation, oocyte meiosis, and cellular senescence (Fig. 4J).

### Integrated transcriptomics and proteomics analysis to identify key regulators of OS-impaired osteogenic differentiation in hBMSCs

**Fig. 5:**
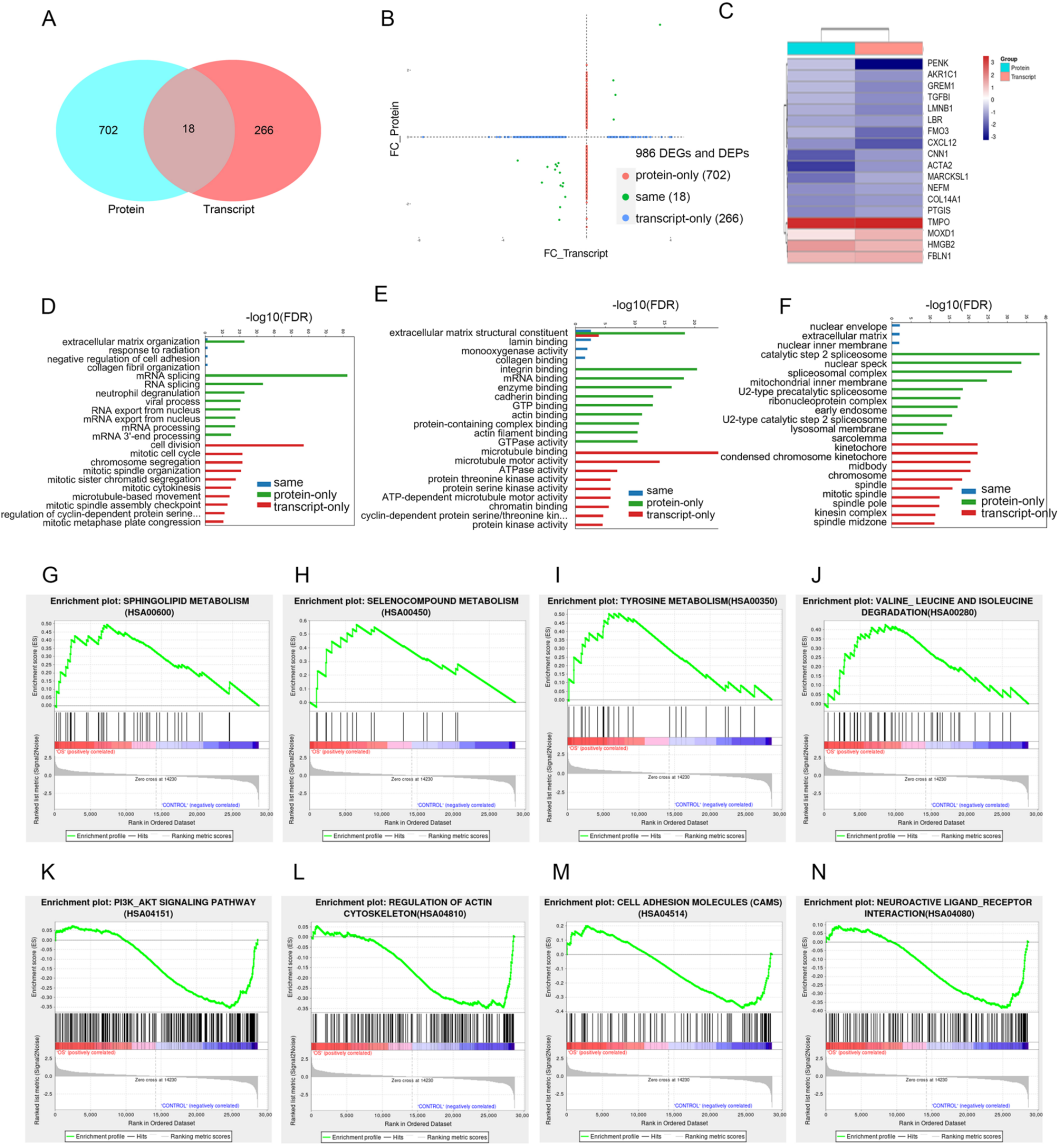
Comprehensive multi-omics analyses of the impaired osteogenic differentiation of hBMSCs under OS. **A**. Venn diagram of differentially expressed molecules in transcriptomic and proteomic datasets (*n* = 3). **B**. Correlation scatter plot with quadrant division of transcriptomic and proteomic expression (*n* = 3). **C**. Integrated clustering heatmap of differentially expressed proteins and their associated transcripts (*n* = 3). **D–F**. GO enrichment analysis of transcriptomic DEGs identified through multi-omics screening (*n* = 3). **G–J**. GSEA reveals significant enrichment of upregulated signaling pathways in BMSCs under OS (*n* = 3). **K–N**. GSEA reveals significant enrichment of downregulated signaling pathways in BMSCs under OS (*n* = 3). hBMSCs: human bone marrow mesenchymal stromal cells; OS: oxidative stress; DEGs: differentially expressed genes; DEPs: differentially expressed proteins; GSEA: gene set enrichment analysis.

We conducted an integrated transcriptomic and proteomic analysis to clarify the mechanism by which OS impairs osteogenic differentiation in hBMSCs. The overlap of significantly altered molecules is shown in Fig. 5A. Correlation analysis revealed a positive concordance in log_2_ fold changes between the two omics layers. The total of 986 differentially expressed molecules identified across both datasets, 702 were significant only at the proteomic level, and 266 were significant only at the transcriptomic level (Fig. 5B), with key targets (|transcriptomic log_2_FC| > 1, |proteomic log_2_FC| > 0.263) systematically regulated at both levels. Unsupervised hierarchical clustering of these concordant molecules defined distinct functional modules responsive to OS (Fig. 5C). This process identified a core set of 18 genes/proteins (PENK, AKR1C1, GREM1, TGFBI, LMNB1, LBR, FMO3, CXCL12, CNN1, ACTA2, MARCKSL1, NEFM, COL14A1, PTGIS, TMPO, MOXD1, HMGB2, and FBLN1) with significant and consistent expression changes across both datasets.

To gain insight into the biological functions of these 18 hub genes, GO annotation and enrichment analyses were performed. The results revealed significant enrichment in pathways associated with dysregulation of the ECM–nucleus signaling axis, alterations in cell adhesion, and remodeling of the extracellular microenvironment, indicating their involvement in stress response and differentiation impairment (Fig. 5G). Specifically, the enriched biological processes included ECM organization, response to radiation, negative regulation of cell adhesion, and collagen fibril organization (Fig. 5D). Enriched molecular functions involved ECM structural constituents, lamin binding, monooxygenase activity, and collagen binding (Fig. 5E). The associated cellular components were mainly localized to the nuclear envelope, extracellular matrix, and nuclear inner membrane (Fig. 5F).

To elucidate the mechanism underlying the impaired osteogenic capacity of hBMSCs under OS, we performed Gene Set Enrichment Analysis (GSEA). The results indicated that multiple metabolic pathways were significantly upregulated, including sphingolipid metabolism, selenocompound metabolism, tyrosine metabolism, and valine/leucine/isoleucine degradation (Fig. 5G–J). In contrast, several signaling pathways were markedly downregulated, such as the PI3K-AKT signaling pathway, regulation of actin cytoskeleton, cell adhesion molecules, and neuroactive ligand-receptor interaction (Fig. 5K–N). Notably, sphingolipid metabolism exhibited the most significant enrichment among all the altered pathways.

### PENK promotes osteogenic differentiation under OS

Among the 18 hub genes identified through integrated transcriptomic and proteomic analysis, PENK was found to be significantly upregulated in response to OS. As a critical endogenous regulator and potential therapeutic target in BMSCs, PENK has been reported to preserve stemness, enhance proliferative capacity, and maintain cellular homeostasis, while also promoting osteogenic differentiation and inhibiting apoptosis in bone-forming cells^16–18^.

**Fig. 6:**
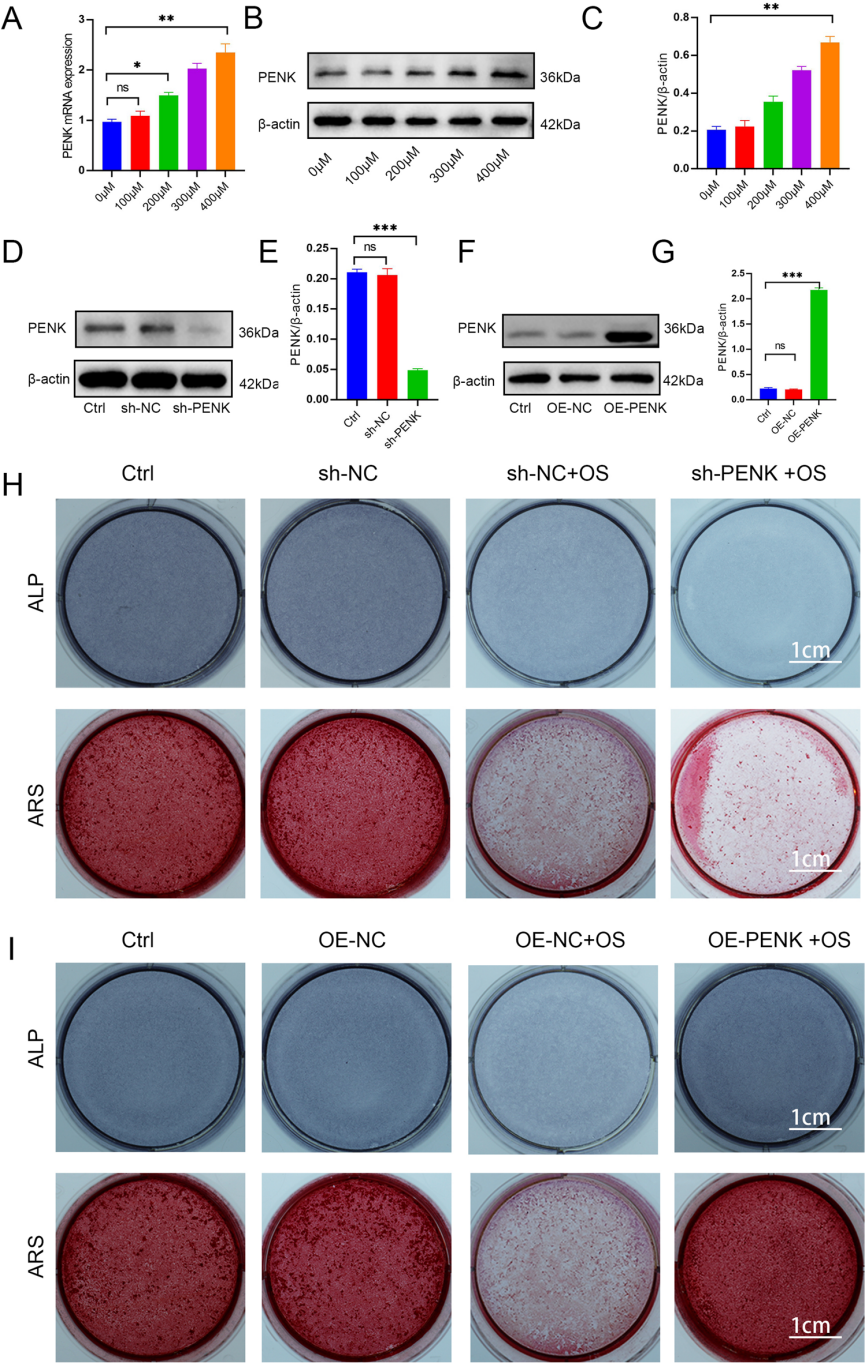
PENK contributes to the osteogenic differentiation of hBMSCs **A.** The expression levels of PENK were detected by RT-qPCR (*n* = 3). **B–G**. The expression levels of PENK were detected by western blotting (*n* = 3). **H**. ALP staining of BMSCs following PENK knockdown and overexpression (*n* = 3). **I**. ARS staining of BMSCs following PENK knockdown and overexpression (*n* = 3). PENK: Proenkephalin; hBMSCs: human bone marrow mesenchymal stromal cells; ALP: alkaline phosphatase staining; ARS: alizarin red S staining. Data are shown as the mean ± SD. *P < 0.05, **P < 0.01, ***P < 0.001, NS, not significant, by one-way ANOVA.

To validate PENK’s regulatory role under OS conditions, hBMSCs were exposed to increasing concentrations of H_2_O_2_ (0, 100, 200, 300, 400 µM). The results revealed a dose-dependent upregulation of PENK expression in response to rising H_2_O_2_ levels (Fig. 6A–C). To further dissect the functional role of PENK, we genetically manipulated its expression in hBMSCs using short hairpin RNA (sh-PENK) to knock down PENK, and lentiviral vectors to achieve PENK overexpression (OE-PENK) (Fig. 6D–G).

Following 400 µM H_2_O_2_ treatment, hBMSCs were subjected to osteogenic induction, and differentiation capacity was evaluated via ALP and ARS staining. The results showed that OS significantly reduced osteogenic differentiation in the control group (sh-NC). Notably, PENK knockdown (sh-PENK) further exacerbated the impairment, resulting in even weaker osteogenic capacity under OS (Fig. 6H). In contrast, PENK overexpression (OE-PENK) significantly enhanced osteogenic differentiation in hBMSCs under the same oxidative conditions (Fig. 6I).

Together, these findings demonstrate that PENK acts as a protective factor in hBMSCs exposed to OS. OS triggers the protective upregulation of PENK, which helps maintain osteogenic potential. Furthermore, PENK overexpression effectively rescues osteogenic differentiation capacity under OS conditions, while PENK silencing aggravates differentiation impairment. These results highlight PENK as a promising target for enhancing bone regeneration under OS.

## Discussion

BMSCs play a central role in bone regeneration through their capacity for osteogenic differentiation, paracrine signaling, and extracellular matrix synthesis. As pivotal seed cells in tissue-engineered bone constructs, BMSCs have demonstrated significant therapeutic potential in improving osteonecrosis and enhancing fracture healing^19–21^. However, their clinical efficacy is often compromised by several factors, notably the OS microenvironment, which markedly impairs their differentiation capacity^22^. Previous studies have shown that OS significantly suppresses the osteogenic differentiation of hBMSCs^23,24^, thereby posing a major obstacle to advancing BMSC-based bone regeneration strategies.

The establishment of a robust experimental model is essential for elucidating these mechanisms. Currently, several agents, including H_2_O_2_^25,26^, test-butyl hydrogen peroxide (TBHP)^27,28^, and deferoxamine (DFO)^29^, are widely used to simulate OS. Among these, H_2_O_2_ is the most frequently used inducer, with effective concentrations typically ranging from 100 µM to 1 mM. The optimal dose for inducing OS without causing complete cell death is approximately 200–550 µM^25,26,30^. In our study, we found that H_2_O_2_ concentrations ≤ 400 µM effectively induced OS while preserving cell viability. In contrast, concentrations ≥ 500 µM resulted in pronounced cytotoxic effects. This enabled us to establish a stable model of OS in hBMSCs and to simulate impaired osteogenic differentiation in vitro.

OS leads to DNA and RNA damage in BMSCs, in addition to promoting protein oxidation and lipid peroxidation. Furthermore, OS influences post-translational modifications in BMSCs, including phosphorylation, glycosylation, methylation, and lactylation. In our transcriptomic analysis, 284 genes were differentially expressed in hBMSCs exposed to OS, predominantly involved in chromosome segregation, nuclear division, organelle fission, cytoskeletal organization, and protein kinase activity. Complementary proteomic analysis revealed 720 differentially expressed proteins associated with disruptions in biological processes, molecular functions, and cellular components. By integrating transcriptomic and proteomic analyses, we identified 18 key genes with consistent differential expression at both mRNA and protein levels, including PENK, AKR1C1, GREM1, TGFBI, LMNB1, LBR, FMO3, CXCL12, CNN1, ACTA2, MARCKSL1, NEFM, COL14A1, PTGIS, TMPO, MOXD1, HMGB2, and FBLN1. These genes primarily function in cell proliferation, differentiation, adhesion, cell cycle regulation, and extracellular matrix composition. Together, these findings indicate that OS impairs essential cellular functions and significantly compromises the osteogenic potential of hBMSCs.

PENK encodes an endogenous opioid precursor and was previously associated with osteoblast differentiation. The human PENK gene was first isolated by Legon et al. in 1982^31^, revealing its molecular structure. Subsequent studies by Rosen et al. indicated that PENK expression plays a role in regulating osteoblast development^32^. Seitz et al. reported that PENK is expressed in osteoblasts and that its deletion in Hyp mice partially rescued bone mineralization defects^33^. However, its function in hBMSC osteogenesis remained largely unexplored. Our data reveal that PENK is upregulated in H_2_O_2_-injured hBMSCs with compromised osteogenic capacity. Lentiviral functional studies confirmed the pro-osteogenic role of PENK overexpression, demonstrated through elevated ALP activity, accelerated calcium deposition, and induction of key osteogenic markers.

Although PENK is traditionally recognized for its role in classical opioid receptor signaling^34^, our multi-omics analysis reveals its unconventional role in regulating osteogenic differentiation. Collectively, we propose that PENK exerts its pro-osteogenic effect by serving as a critical metabolic switch that acts through sphingolipid metabolism, thereby linking oxidative stress responses to cell fate determination in hBMSCs.

While our findings show promise, certain limitations merit consideration: (1) The dependency on transcriptomic/proteomic screening—though identifying PENK as a key OS-responsive regulator of osteogenesis—lacks in vivo validation of its bone regenerative therapeutic potential; (2) Putative signaling pathways underlying PENK-mediated osteogenesis, identified via multi-omics, require direct mechanistic confirmation through pathway perturbation and receptor interaction studies. These gaps necessitate future work to fully delineate PENK’s molecular machinery and clinical translatability. We emphasize that these aspects constitute active priorities for ongoing investigation.

In conclusion, this study establishes an OS-induced model of impaired osteogenic differentiation in hBMSCs and provides mechanistic insights into the regulatory networks involved. Through integrated transcriptomic and proteomic profiling, we identified 18 key genes implicated in osteogenic dysfunction, with PENK emerging as a novel positive regulator of hBMSC osteogenesis. These findings not only deepen our understanding of hBMSC biology under oxidative conditions but also propose PENK as a promising biomarker and therapeutic target for skeletal disorders such as osteonecrosis and bone defects.

## Materials and methods

### Ethics

All human-related scientific experiments were approved by the Medical Ethics Committee of the Affiliated Hospital of Guizhou Medical University (Grant No. 2025-40). All methods were carried out in accordance with relevant guidelines and regulations. Informed consent was obtained from all subjects and/or their legal guardians. All research involving human participants was conducted in accordance with the Declaration of Helsinki.

### Clinical samples

This study involved the extraction of hBMSCs from bone marrow tissues obtained from hip replacement patients at the Affiliated Hospital of Guizhou Medical University (Guiyang, Guizhou 550004, China). Patients who underwent hip replacement surgery at the hospital between July 2023 and July 2024 were included based on the following criteria: (1) age between 45 and 70 years and (2) scheduled hip replacement surgery. Exclusion criteria included: (1) hematological disorders, (2) bone metabolism diseases or long-term medication affecting bone metabolism, (3) severe cardiopulmonary diseases, and (4) hereditary diseases. All participants were informed about the study procedures, and they provided written informed consent.

### Sample collection and processing

After obtaining informed consent, we collected clinical data from 9 patients diagnosed with traumatic femoral neck fracture (Garden types III-IV). Bone marrow and medullary cavity tissues, which would otherwise have been discarded during hip replacement surgeries, were harvested as samples. The extracted hBMSCs were pooled after extraction and expansion, divided into 6 equal portions, and randomly assigned to OS and control groups using an unpaired experimental design. Transcriptomic and proteomic sequencing was subsequently performed to minimize potential confounding effects of age and gender differences on the experimental outcomes, as detailed in Supplementary Table 1.

### hBMSCs isolation and culture

Bone marrow and medullary cavity tissues were processed for hBMSCs extraction using the Ficoll density gradient separation method. The bone marrow cavity was flushed with complete Dulbecco’s Modified Eagle Medium (DMEM) (Gibco, USA, C11885500BT), supplemented with 10% fetal bovine serum (Gibco, USA, A5256701), 100 U/mL penicillin, and 100 µg/mL streptomycin (Hyclone, USA, 10378016), to obtain a bone marrow cell suspension. Cells were cultured at 37 °C in a 5% CO_2_ atmosphere. Once cells reached 90% confluence, they were enzymatically dissociated using 1 mL of a solution containing 0.25% trypsin and 0.02% ethylenediaminetetraacetic acid (EDTA, USA, 25200056) at 37 °C. The cells were then subcultured at a 1:3 ratio.

### Flow cytometry

Third-passage hBMSCs were detached using 0.25% trypsin-0.02% EDTA, washed twice with phosphate-buffered saline (PBS), and resuspended at a density of 2 × 10⁷ cells/mL in staining buffer. For surface marker analysis, 1 × 10⁶ cells were aliquoted per tube. The following antibody staining scheme was used: an unstained control (buffer only); single-stained controls for each marker; and a test sample stained with a cocktail of all antibodies. All antibodies were used at manufacturer-recommended concentrations and were purchased from BD Biosciences (USA): CD90 (561971), CD105 (566265), CD34 (550619), and CD45 (560915). Cells were incubated with antibodies for 20 min at room temperature in the dark, washed twice with staining buffer, and resuspended in 500 µL of buffer for analysis. Data were acquired using a BD FACSCanto II flow cytometer and analyzed with FlowJo v10.8 software.

### hBMSCs model of OS

Third-generation hBMSCs were randomly allocated to either the experimental group (OS) or the control group. The experimental group was treated with a range of H_2_O_2_ concentrations (100, 200, 300, 400, 500, and 600 µM) for 6 days. Cellular morphology and viability were assessed microscopically, and cell viability was quantified using the Cell Counting Kit-8 (CCK-8) assay (Beyotime, China, C0038).

### Mitochondrial membrane potential detection

hBMSCs were treated with different H_2_O_2_ concentrations for 24 h, after which the JC-1 mitochondrial membrane potential detection kit (KeyGen Biotech, China, KGA604) was used. The cells were then incubated at 37 °C for 20 min, followed by imaging with a laser confocal microscope.

### ROS determination

hBMSCs were treated with different H_2_O_2_ concentrations for 24 h, after which DHE (Sigma, USA, S0064S) was diluted 1:1000 in the culture medium and used to treat cells at 37 °C for 20 min, followed by imaging with a laser confocal microscope.

### Osteogenic differentiation of hBMSCs

Third-generation hBMSCs were seeded in 6-well plates. Upon reaching 60%–70% confluence, the experimental group was treated with osteogenic-differentiation medium according to the protocol of the OriCell^®^ human BMSC osteogenic induction differentiation kit (Cyagen, China, HUXMX-90021), while the control group was maintained in complete DMEM. After 1 week of induction, alkaline phosphatase (ALP) activity, an early marker of osteogenic differentiation, was assessed using ALP staining (Beyotime, China, C3206). Following 3 weeks of induction, matrix mineralization and calcium nodule formation were evaluated by Alizarin Red S (ARS) staining (Cyagen, China, OILR-10001).

### Chondrogenic differentiation of hBMSCs

Third-generation hBMSCs were seeded in 6-well plates. Upon reaching 80% confluence, the experimental group was treated with chondrogenic-differentiation medium following the manufacturer’s protocol for the OriCell^®^ human BMSC chondrogenic induction differentiation kit (Cyagen, China, HUXMX-90041), while the control group was maintained in complete DMEM. The chondrogenic medium was refreshed every 2–3 days. Following chondrogenic induction for 3 weeks, the cells were fixed, and sulfated proteoglycans in the extracellular matrix were identified via Alcian blue staining (Cyagen, China, ALCB-10001).

### Adipogenic differentiation of hBMSCs

After seeding third-generation hBMSCs in 6-well plates, the cells were cultured until they reached 80% confluence. The experimental group was then treated with adipogenic-differentiation medium, following a cyclic protocol in accordance with the instructions of the OriCell^®^ human BMSC adipogenic induction differentiation kit (Cyagen, China, HUXMX-90031). cells were first exposed to Adipogenic Induction Medium (Solution A) for 3 days, followed by Adipogenic Maintenance Medium (Solution B) for 1 day. This cycle was repeated until abundant lipid droplets were observed. The control group was maintained in complete DMEM. Following complete adipogenic induction, the cells were fixed, and intracellular neutral lipid droplets were identified via Oil Red O staining (Cyagen, China, OILR-10001).

### Modeling of dysregulated hBMSCs osteogenic differentiation in OS

Third-generation hBMSCs were randomly assigned to OS and control groups. The experimental OS group was treated with H_2_O_2_ at concentrations of 0, 200, 300, or 400 µM for 6 days, after which osteogenic induction was initiated. ALP staining was conducted after a 1-week period, followed by ARS staining after 3 weeks.

#### Transcriptomic sequencing

Third-generation hBMSCs were categorized into two experimental groups: a control group and an OS group, with each group comprising three biological replicates (*n* = 3). Total RNA was extracted using Trizol reagent, and following a comprehensive quality assessment, RNA sequencing (RNA-seq) was performed. Complementary DNA (cDNA) was synthesized from the extracted RNA, followed by cDNA end repair, adenine overhang addition (A-tailing), adapter ligation, and RT-qPCR amplification to construct the sequencing library. Sequencing was carried out using the Illumina NovaSeq platform. The raw FASTQ data underwent preprocessing using proprietary Perl scripts, with stringent quality control measures implemented before data acquisition.

Differential expression analysis was conducted using the DESeq2 R package (version 1.16.1) to compare the hBMSCs-CONTROL and hBMSCs-OS groups (*n* = 3). The Benjamini-Hochberg method was applied to adjust P-values, with genes exhibiting an adjusted P-value less than 0.05 considered as differentially expressed genes. Subsequent enrichment analyses for GO terms and KEGG pathways^35,36^were performed using the ClusterProfiler R package, identifying significantly enriched GO terms with an adjusted P-value < 0.05.

### Proteomic sequencing

Third-generation hBMSCs were divided into control and OS groups (*n* = 3). Protein samples were first lysed using SDT lysis buffer (4% SDS, 100 mM Tris-HCl, 100 mM DTT, pH 7.6). Samples were sonicated and boiled at 100 °C for 15 min, followed by centrifugation at 14,000 g for 15 min. The supernatants were collected, and protein concentrations were determined using the BCA assay. Protein integrity was assessed by SDS-PAGE. For each sample, 20 µg of protein was mixed with 6× loading buffer, boiled for 5 min, and loaded onto a 12% SDS-PAGE gel. For digestion, the FASP (Filter-Aided Sample Preparation) method was used. Briefly, 50–200 µg of protein was reduced with 100 mM DTT (boiled for 5 min), then mixed with UA buffer (8 M urea in 150 mM Tris-HCl, pH 8.5) and transferred to 30 kDa ultrafiltration units. After centrifugation and buffer exchange, proteins were alkylated with 100 mM IAA in UA buffer in the dark for 30 min, followed by repeated washes with UA buffer and 50 mM ammonium bicarbonate. Proteins were digested with trypsin (1:50, w/w) in 40 mM ammonium bicarbonate at 37 °C for 16–18 h. Peptides were recovered by centrifugation, desalted using C18 cartridges, lyophilized, and reconstituted in 0.1% formic acid for LC-MS/MS analysis. Peptide concentration was measured by OD280.

Proteomic analysis was conducted using a nanoElute system coupled with a timsTOF Pro (Bruker) in PASEF mode. The mass range was set at 100–1700 m/z, with a 1/K0 range of 0.75–1.4 V·s/cm², a ramp time of 100 ms, and a 100% lock duty cycle. Additional parameters included a capillary voltage of 1500 V, a dry gas flow rate of 3 L/min, a dry temperature of 180 °C, 10 MS/MS scans per cycle (total cycle time: 1.16 s), a charge range of 0–5, active exclusion for 0.5 min, a target intensity of 10,000, and an intensity threshold of 2500, with CID settings of 20–59 eV.

MS data analysis was performed using MaxQuant v1.6.17.0 with a project-specific database. The initial search employed a 6 ppm precursor mass window, following Trypsin/P cleavage rules, allowing up to two missed cleavages, and a 20 ppm mass tolerance for fragment ions. Carbamidomethylation of cysteines was set as a fixed modification, while protein N-terminal acetylation and methionine oxidation were considered variable modifications. The global false discovery rate (FDR) for peptide and protein identification was set at 0.01. Protein abundance was determined using normalized spectral protein intensity (LFQ intensity). Proteins with a fold change greater than 2 or less than 0.5 and a P-value below 0.05 (Student’s *t*-test) were classified as differentially expressed proteins.

#### Proteomic mass spectrometry and data analysis

The study utilized the UniProt database (uniprot-homo-20231008-20427-9606-swiss-prot), accessible at http://www.uniprot.org. Data analysis was performed using Perseus v1.3 (Max Planck Institute of Biochemistry, Martinsried, Germany), MaxQuant v1.6.17.0, and R v3.3.1. The GO database version used for these analyses was go.obo (2019-07-01), while the KEGG database version was KO-INFO-END.txt (2023-10-17). Bioinformatics analysis was performed using Metascape (version 3.5.20250101; https://metascape.org/), an integrated resource for gene annotation and pathway enrichment. Protein-protein interaction (PPI) network analysis results were constructed using AnyChart software (Version 8.11.0.1934).

#### Correlation analyses

To integrate transcriptomic and proteomic datasets, quantitative detection results from both domains were analyzed to identify overlapping differentially expressed mRNAs and proteins. These analyses included correlation analysis, association clustering, and GO and KEGG pathway enrichment approaches.

#### Real-time quantitative polymerase chain reaction (RT-qPCR)

Total RNA was extracted from bone tissue or cultured cells using TRIzol (Invitrogen, USA, 15596026). Bone tissue was frozen with liquid nitrogen, ground into powder, and subjected to RNA isolation. For cells, RNA extraction was performed directly by lysing them with TRIzol. The RNA was purified using the RNAeasy kit (Sangon Biotech), quantified with a Nanodrop spectrophotometer, and converted to cDNA using the PrimeScriptTM RT kit (Sangon Biotech, China, B532431). RT-qPCR was conducted with the SYBR Premix Ex Taq II kit (Sangon Biotech, China, B110032) on an Applied Biosystems 7500 system. GAPDH served as the reference gene, and relative transcript levels were analyzed using the 2-∆∆Ct method. The primers (Sangon Biotech, China) were as follows: OPN-F: AAT CTC CTA GCC CCA CAG ACC; OPN-R: CAC ACT ATC ACC TCG GCC ATC; RUNX2-F: TAG GCG CAT TTC AGG TGC TT; RUNX2-R: TGG CAG GTA GGT GTG GTA GT; PENK-F: AAG TGA GAT CCT CGC CAA GC; PENK-R: AGC AGG TCT GAG GAA TTG GC.

### Western blotting

Proteins were extracted using 1× cell lysis buffer (Beyotime, China, P0013B), separated by 10% or 12% SDS-PAGE (Beyotime, China, P0012AC), and transferred to PVDF membranes (Thermo Scientific, USA, 88518). Western blotting was performed using an ECL chemiluminescence reagent (Merck Millipore, China, WBKLS0100). Primary antibodies were acquired from the following sources: β-actin (1:3000, 81115-1-RR), RunX2 (1:1000, ab192256), and OPN (1:1000, ab75285) were from Abcam (USA); PENK (1:1000, A6302) was from Abclonal Technology (China). An anti-IgG secondary antibody (1:2000, Abcam, USA, ab172273) was used for detection. Protein bands were visualized using a gel imaging system (Clinx Science Instruments, Shanghai, China) and quantified with ImageJ software (v1.4.3.67, National Institutes of Health, USA).

### Lentiviral vector production and cell transfection

The lentiviral (LV) vectors used for knocking down or overexpressing PENK were obtained from Genechem (Shanghai, China). Specifically, the PENK-RNAi constructs (130538/130539/130540) and the overexpression vector (Oe PENK, KL3327-1) were designed based on distinct regions of the human PENK gene. A control LV vector containing a nonspecific RNA oligonucleotide (sh-NC or Oe-NC) was employed as a negative control.

For cell transfection, hBMSCs (third generation) were transfected with LV vectors at a multiplicity of infection (MOI) of 20. After 24 h, the medium was replaced with complete DMEM. Three days post-infection, transfection efficiency was assessed by GFP expression under an inverted fluorescence microscope. After 6 days, the cells were selected in complete medium containing 2 µg/mL puromycin (Solarbio, Beijing, China), and PENK expression was evaluated using RT-qPCR.

### ALP staining

Cells cultured in 6-well plates or other containers were washed with phosphate-buffered saline (PBS) (VivaCell, China, C3580), and the medium was discarded. ALP staining solution was added using the azo coupling method, and the cells were fixed for 3 min, or they were fixed using 4% paraformaldehyde for 10–15 min, followed by washing with PBS. The prepared ALP staining solution was then applied (azo coupling method), and samples were incubated in a dark, humid chamber for 15–20 min. The staining solution was removed, and the cells were rinsed gently with PBS. For contrast, nuclei were counterstained with nuclear fast red for 5 min. After a final PBS wash, the staining results indicative of early osteogenic activity were examined and recorded under a light microscope.

### Alizarin red staining

After 3 weeks of osteogenic induction, the complete osteogenic differentiation medium was removed from the 6-well plates, and each well was gently washed 2–3 times with 1× PBS. Next, 2 mL of 4% paraformaldehyde was added to each well, and the cells were fixed at room temperature for 30 min. The fixation solution was then discarded, and the cells were washed 2–3 times with 1× PBS. Next, 2 mL of Alizarin Red working solution was added to each well, and the cells were stained at room temperature for 5–10 min. The Alizarin Red staining solution was then removed, and the cells were washed gently with 1× PBS 2–3 times to completely eliminate excess dye. After adding 2 mL of 1× PBS to each well, osteogenic staining was observed under a microscope. The stained 6-well plates were then sealed with a sealing film and stored at 4 °C for up to 2 weeks.

### Oil red O staining

After 3 weeks of adipogenic induction, to detect intracellular lipid droplets, the cells were washed with PBS and fixed with 4% paraformaldehyde for 15 min at room temperature. Following fixation, the cells were washed twice with PBS. Subsequently, 500 µL of filtered Oil Red O working solution was added to each well, and the plates were incubated for 20 min at room temperature. After incubation, the staining solution was removed, and the cells were gently washed twice with PBS to remove non-specific background. Stained lipid droplets were then visualized and photographed under a light microscope.

#### Alcian blue staining

After 3 weeks of chondrogenic induction, to detect sulfated proteoglycans in the extracellular matrix, the cells were washed with PBS and fixed with 4% paraformaldehyde for 15 min at room temperature. After removing the fixative, the cells were washed twice with PBS. Then, 500 µL of Alcian blue staining solution (pH 2.5) was added to each well, and the plates were incubated for 20 min at room temperature. Following incubation, the staining solution was aspirated, and the cells were rinsed twice with PBS. The blue-stained matrix was examined and recorded under a light microscope.

### Statistical analysis

All data were analyzed and visualized using GraphPad Prism 9.0. In vitro experiments included at least three biological replicates, while in vivo experiments had at least five. The Shapiro-Wilk test assessed normality, and the Levene test evaluated variance homogeneity. Data following a normal distribution were presented as mean ± standard deviation, while non-normally distributed data were expressed as the median or interquartile range. Statistical comparisons were performed using unpaired t-tests for two groups and one-way ANOVA with Tukey’s post-hoc test for multiple groups. For non-normally distributed or heteroscedastic data, the Wilcoxon rank-sum test was used for two-group comparisons, and the Kruskal-Wallis H test was used for multiple-group comparisons. A P-value < 0.05 was considered statistically significant.

## Supplementary Information

Below is the link to the electronic supplementary material.

Supplementary material 1 (JPG 1313.3 kb)

Supplementary material 2 (JPG 394.6 kb)

Supplementary material 3 (DOCX 17.1 kb)

Supplementary material 4 (DOCX 15.9 kb)

## Data Availability

The transcriptomics dataset has been deposited in the NCBI GEO repository under accession number GSE318199. The mass spectrometry proteomics data have been deposited to the ProteomeXchange Consortium via the PRIDE^37^ partner repository with the dataset identifier PXD067569. All data generated or analyzed during this study are included in this published article. All data and reagents are available from the corresponding author upon reasonable request.
