# Supplementary figures and images for: Multi-omics identification of key targets for the osteogenic differentiation of human bone marrow mesenchymal stromal cells under oxidative stress
Source: Sci Rep. 2026 Feb 10;16:8215. doi: 10.1038/s41598-026-39818-4 (PMC12963487; doi:10.1038/s41598-026-39818-4)

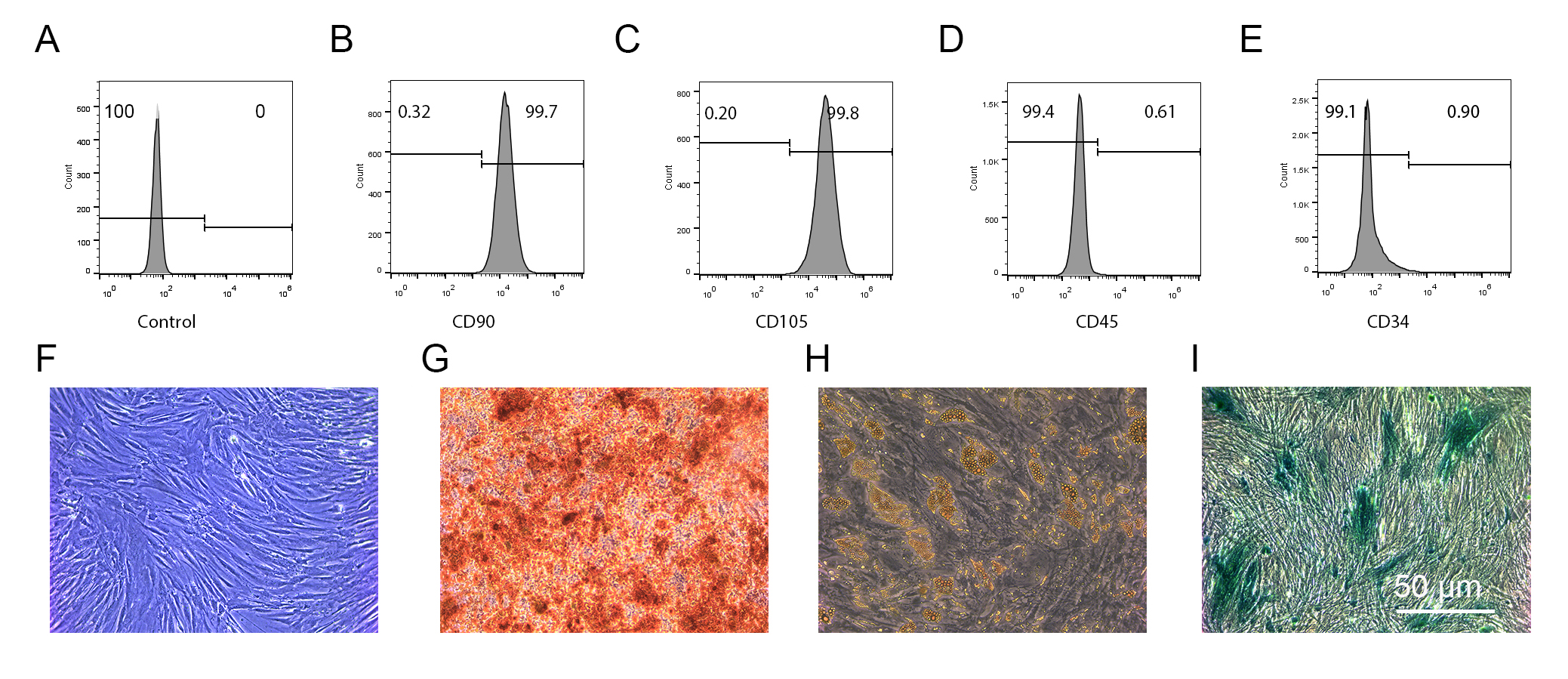

Supplement: Supplementary file 1 — Supplementary material 1 (JPG 1313.3 kb) [file 41598_2026_39818_MOESM1_ESM.jpg]

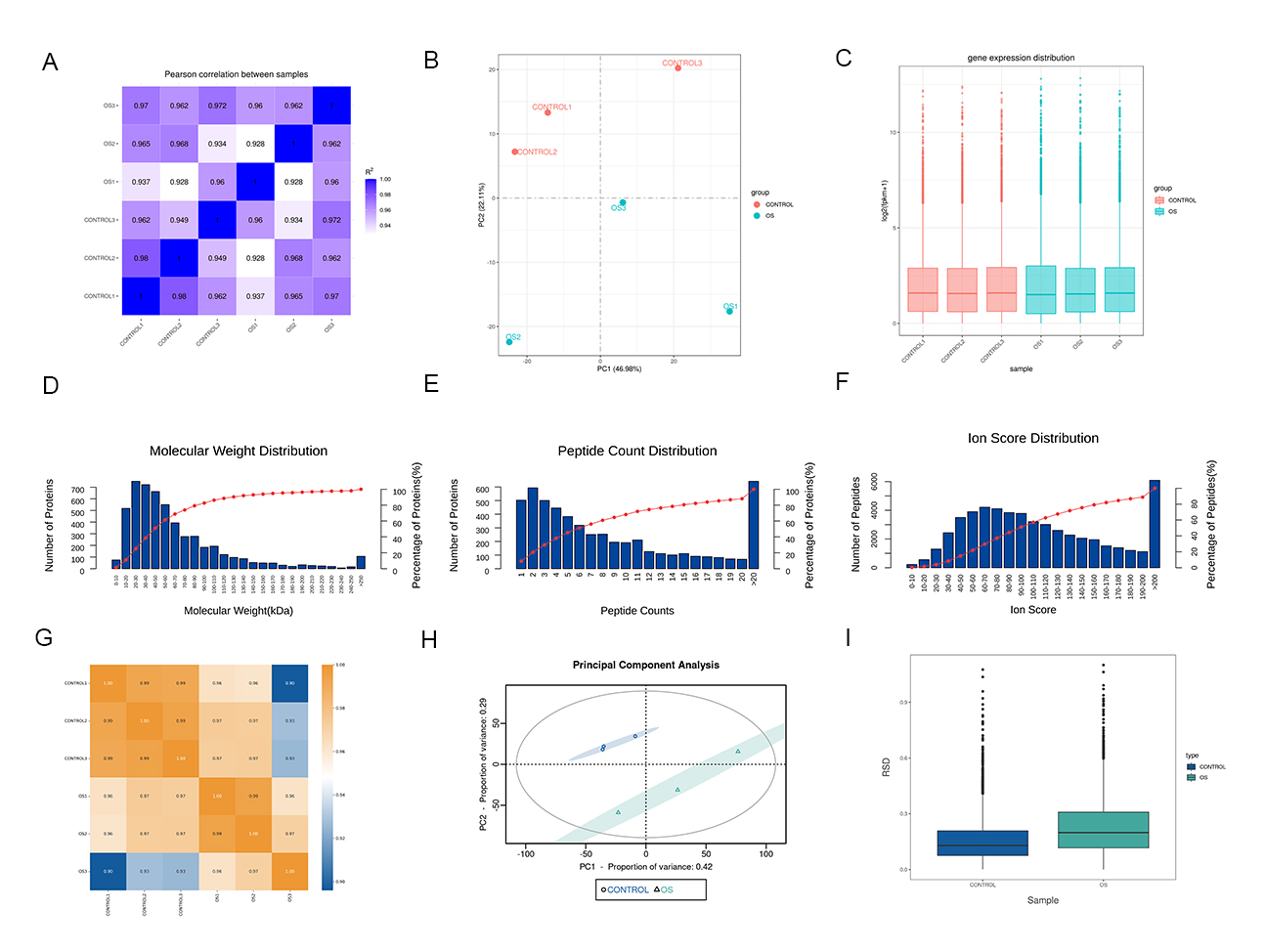

Supplement: Supplementary file 2 — Supplementary material 2 (JPG 394.6 kb) [file 41598_2026_39818_MOESM2_ESM.jpg]
