# Supplementary material for: Multi-omics identification of key targets for the osteogenic differentiation of human bone marrow mesenchymal stromal cells under oxidative stress
Source: Sci Rep. 2026 Feb 10;16:8215. doi: 10.1038/s41598-026-39818-4 (PMC12963487; doi:10.1038/s41598-026-39818-4)
Supplement: Supplementary file 3 — Supplementary material 3 (DOCX 17.1 kb) [file 41598_2026_39818_MOESM3_ESM.docx]

**Supplementary Figure legends**

**Supplementary Fig. 1** Characterization and tri-lineage differentiation of hBMSCs

1. Control group.

B–C. hBMSCs exhibited typical MSC markers (CD90⁺, CD105⁺) (n = 3).

D–E. hBMSCs lacked hematopoietic markers (CD45⁻, CD34⁻) (n = 3).

F–I. hBMSCs showed typical spindle-like morphology, demonstrated osteogenic, adipogenic, and chondrogenic differentiation capacity (n = 3). F: control; G: osteogenic differentiation; H: adipogenic differentiation; I: chondrogenic differentiation.

**Supplementary Fig. 2** Transcriptomic and proteomic data quality control

1. Correlation heatmap of transcriptomic samples (n = 3).
2. PCA of transcriptomic samples (n = 3).
3. Boxplot of transcriptomic samples (n = 3).
4. Distribution of identified protein molecular weights (n = 3).
5. Distribution of the number of identified peptides per protein (n = 3).
6. Distribution of peptide ion scores in proteomic analysis (n = 3).
7. Pearson correlation analysis of proteomic samples (n = 3).
8. PCA of proteomic samples (n = 3).
9. RSD analysis of proteomic data (n = 3).

PCA: Principal component analysis; RSD: Relative standard deviation.
