# Supplementary material for: Multi-omics identification of key targets for the osteogenic differentiation of human bone marrow mesenchymal stromal cells under oxidative stress
Source: Sci Rep. 2026 Feb 10;16:8215. doi: 10.1038/s41598-026-39818-4 (PMC12963487; doi:10.1038/s41598-026-39818-4)
Supplement: Supplementary file 4 — Supplementary material 4 (DOCX 15.9 kb) [file 41598_2026_39818_MOESM4_ESM.docx]

Supplementary Table 1. Clinical characteristics of patients and experimental usage

| **Patient ID** | **Gender** | **Age** | **Transcriptom** | **Proteomics** | **ROS** | **MMP** | **ALP** | **ARS** | **RT-qPCR** | **WB** |
| --- | --- | --- | --- | --- | --- | --- | --- | --- | --- | --- |
|  |  | **(years)** |  |  |  |  |  |  |  |  |
| Patient 1 | F | 67 | – | – | √ | √ | √ | √ | √ | √ |
| Patient 2 | F | 47 | – | – | √ | √ | √ | √ | √ | √ |
| Patient 3 | M | 60 | – | – | √ | √ | √ | √ | √ | √ |
| Patient 4 | F | 46 | √ | √ | √ | √ | √ | √ | √ | √ |
| Patient 5 | M | 45 | √ | √ | √ | √ | √ | √ | √ | √ |
| Patient 6 | M | 57 | √ | √ | √ | √ | √ | √ | √ | √ |
| Patient 7 | F | 63 | √ | √ | √ | √ | √ | √ | √ | √ |
| Patient 8 | F | 58 | √ | √ | √ | √ | √ | √ | √ | √ |
| Patient 9 | F | 67 | √ | √ | √ | √ | √ | √ | √ | √ |

Note：hBMSCs were pooled, divided into six aliquots, and randomly assigned to control and OS groups for transcriptomic and proteomic sequencing to control for donor age and sex differences.

ROS: reactive oxygen species; MMP: mitochondrial membrane potential; ALP: alkaline phosphatase staining; ARS: alizarin red S staining; RT-qPCR: real-time quantitative polymerase chain reaction; WB: western blotting; F: female; M: male; √: experiment performed; –: hBMSCs: Human bone marrow mesenchymal stromal cells;not performed; hBMSCs: Human bone marrow mesenchymal stromal cells; OS: oxidative stress.
